# Epilepsy, Cognitive, and Behavioral Outcomes in Neurocutaneous Syndromes: A Comparative Review of NF1, TSC, and Sturge–Weber Syndrome

**DOI:** 10.3390/children13070912

**Published:** 2026-07-09

**Authors:** Aurora Alexandra Jurca, Romana Vulturar, Adina Chis, Ana Lucretia Trandafir, Codruța Diana Petchesi, Kinga Kozma, Emilia Severin, Ramona Hodisan, Claudia Maria Jurca, Simona Ioana Vicas, Sanziana Iulia Jurca, Alexandru Daniel Jurca

**Affiliations:** 1 Faculty of Medicine and Pharmacy, University of Oradea, Universitatii Street 1, 410081 Oradea, Romania; jurca.auroraalexandra@student.uoradea.ro (A.A.J.); trandafir.analucretia@student.uoradea.ro (A.L.T.); jurca.sanzianaiulia@student.uoradea.ro (S.I.J.); 2 Discipline of Cell and Molecular Biology, “Iuliu Hațieganu” University of Medicine and Pharmacy, 6, Pasteur St., 400349 Cluj-Napoca, Romania; vulture.romana@umfcluj.ro (R.V.); achis@umfcluj.ro (A.C.); 3 Department of Preclinical Disciplines, Faculty of Medicine and Pharmacy, University of Oradea, Universitatii Street 1, 410081 Oradea, Romania; codruta.diana.petchesi@didactic.uoradea.ro (C.D.P.); alexjurca@uoradea.ro (A.D.J.); 4 Regional Center of Medical Genetics Bihor (Part of ERN-ITHACA), County Emergency Clinical Hospital Oradea, 410469 Oradea, Romania; 5 Department of Genetics, University of Medicine and Pharmacy “Carol Davila” Bucharest, Dionisie Lupu Street, Number 37, 020021 Bucharest, Romania; 6 Department of Food Engineering, University of Oradea, 26 Gen. Magheru St., 410048 Oradea, Romania; simona.vicas@didactic.uoradea.ro

**Keywords:** neurocutaneous syndromes, epilepsy, neurodevelopment, cognitive impairment, behavioural disorders, neurofibromatosis type 1, tuberous sclerosis complex, Sturge–Weber syndrome, intellectual disability, mTOR, RAS/MAPK signalling

## Abstract

**Highlights:**

**What are the main findings?**

- Early-onset and poorly controlled epilepsy are consistently associated with worse cognitive and behavioral outcomes in tuberous sclerosis complex and Sturge–Weber syndrome and may contribute to neurodevelopmental impairment in neurocutaneous syndromes overall.
- Despite distinct genetic etiologies, neurofibromatosis type 1, tuberous sclerosis complex, and Sturge–Weber syndrome converge on shared molecular pathways and network abnormalities that contribute to epileptogenesis and neurodevelopmental dysfunction.

**What are the implications of the main findings?**

- Recognising epilepsy as an important modifier of neurodevelopmental vulnerability may support earlier intervention and more comprehensive care for children with neurocutaneous syndromes.
- A better understanding of the shared mechanisms linking epilepsy and neurodevelopment may help refine prognostic assessment and guide the development of more targeted therapeutic strategies.

**Abstract:**

Background: Neurocutaneous syndromes (NCS), including neurofibromatosis type 1 (NF1), tuberous sclerosis complex (TSC), and Sturge–Weber syndrome (SWS), are rare neurodevelopmental disorders frequently associated with epilepsy, cognitive impairment, and behavioural difficulties. Although caused by different genetic alterations, these disorders share biological mechanisms that influence brain development, neuronal connectivity, and network excitability. Beyond being a common neurological manifestation, epilepsy is increasingly recognized as an important factor influencing cognitive and behavioural outcomes in neurocutaneous syndromes. Methods: This narrative review summarizes current evidence on the relationship between epilepsy, cognitive dysfunction, and behavioural manifestations in major neurocutaneous syndromes. Attention is given to epileptogenic mechanisms, shared molecular pathways, and factors influencing long-term neurodevelopmental outcomes. Results: Epilepsy is consistently associated with cognitive and behavioural outcomes in neurocutaneous syndromes, particularly in disorders characterized by early-onset and treatment-resistant seizures. Early seizure onset, poor seizure control, and persistent network dysfunction have been associated with intellectual disability, executive dysfunction, attention deficits, autism spectrum features, and impaired adaptive functioning. In TSC and SWS, epilepsy burden is strongly associated with cognitive outcome, particularly in interaction with underlying structural, vascular, and molecular abnormalities. In neurofibromatosis type 1, cognitive and behavioural difficulties are more often related to altered neuronal connectivity and dysregulated signalling pathways, although epilepsy may further contribute to neurodevelopmental impairment in a subset of patients. Despite their distinct genetic origins, these disorders converge on dysregulated RAS/MAPK, PI3K/AKT/mTOR, and Gαq-mediated signalling pathways that influence both epileptogenesis and brain development. Conclusions: Despite their distinct genetic origins, major neurocutaneous syndromes converge on common pathways linking epilepsy, network dysfunction, and neurodevelopmental impairment. Understanding how these processes interact may facilitate earlier intervention and more accurate prognostic assessment, ultimately improving long-term outcomes for affected children.

## 1. Introduction

Neurocutaneous syndromes (NCS), also known as phakomatoses, comprise a heterogeneous group of disorders predominantly affecting tissues of neuroectodermal origin, particularly the skin, central nervous system, and ocular structures. Historically, these syndromes were recognized based on characteristic clinical manifestations; however, advances in molecular genetics and neuroimaging have refined their classification and expanded the understanding of their biological basis by enabling the identification of specific causative alterations [1]. These developments have improved diagnostic accuracy, prognostic assessment, genetic counselling, and therapeutic decision-making, while also facilitating the emergence of targeted treatment approaches [2,3,4].

The most common neurocutaneous syndromes include neurofibromatosis type 1 (NF1), tuberous sclerosis complex (TSC), and Sturge–Weber syndrome (SWS), while conditions such as incontinentia pigmenti, hypomelanosis of Ito, and ataxia telangiectasia are encountered less frequently [5]. These disorders arise from distinct genetic or somatic mosaic alterations that may affect partially overlapping biological pathways involved in neuronal differentiation, synaptic plasticity, cortical organization, vascular development, neuronal excitability, and network connectivity [1,6,7]. As a result, affected individuals may develop a broad spectrum of systemic, neurological, cognitive, behavioural, and adaptive functioning difficulties.

Neurodevelopmental manifestations are common across NF1, TSC, and SWS, but their profiles differ. In NF1, learning difficulties, attention deficits, executive dysfunction, visuospatial impairment, language-related difficulties, and social communication problems are frequently reported, whereas intellectual disability is less common [8,9,10,11]. In TSC, neurodevelopmental impairment is often broader and may include intellectual disability, autism spectrum features, attention-deficit/hyperactivity disorder, sleep problems, anxiety, behavioural dysregulation, academic difficulties, and other manifestations included within the framework of TSC-associated neuropsychiatric disorders [12,13,14]. In SWS, cognitive and behavioural outcomes are highly variable and are influenced by the extent of cerebral involvement, vascular–metabolic injury, epilepsy severity, motor and visual impairment, and adaptive functioning difficulties [15,16].

Epilepsy is also a major neurological manifestation of neurocutaneous syndromes, but its frequency, severity, and prognostic relevance differ substantially across disorders. In NF1, seizures occur in a minority of patients, and cognitive or behavioural difficulties often arise independently of epilepsy [11,17]. In contrast, epilepsy is more frequent and often more severe in TSC and SWS, where early-onset seizures, refractory epilepsy, and persistent network dysfunction may further influence neurodevelopmental trajectories [12,13,14,15,16].

Neuroimaging has contributed substantially to the characterization of central nervous system involvement and systemic manifestations in neurocutaneous syndromes, supporting a more integrated understanding of structural, vascular, and developmental abnormalities [18]. In addition to neurological and cognitive outcomes, patient-reported measures and disease-specific quality-of-life instruments have highlighted the broader functional and psychosocial burden of these disorders, particularly in TSC [19]. To improve transparency in the literature selection process, the present review documents article identification and inclusion using the PRISMA 2020 framework, as detailed in the Materials and Methods section [20]. Diagnostic and molecular advances have also refined syndrome-specific classification, as illustrated by revised diagnostic criteria for NF1 and Legius syndrome [21].

At the molecular level, these disorders illustrate distinct but partially convergent pathogenic mechanisms. Loss of neurofibromin function in NF1 results in dysregulation of RAS/MAPK signalling, whereas pathogenic variants in TSC1 or TSC2 lead to dysregulation of the PI3K/AKT/mTOR signalling cascade [22,23,24]. In SWS, somatic activating mutations of GNAQ promote aberrant Gαq-mediated signalling, with downstream effects on MAPK and PI3K/AKT/mTOR-related pathways [25,26,27].

Although initiated by distinct genetic or somatic alterations, these pathways converge on biological processes that regulate neuronal differentiation, synaptic development, cortical organization, vascular integrity, excitatory–inhibitory balance, and network excitability.

Importantly, neurodevelopmental impairment in these syndromes cannot be attributed to epilepsy alone. NF1, TSC, and SWS are each associated with intrinsic molecular, structural, vascular, and network-level abnormalities that may affect neurodevelopment even in the absence of seizures. However, epilepsy may further compound these pre-existing vulnerabilities, particularly when seizures begin early, are frequent or prolonged, remain drug-resistant, or are associated with persistent epileptiform abnormalities. This interaction appears especially relevant in TSC and SWS, where epilepsy burden is generally greater than in NF1.

Despite increasing knowledge about the clinical and molecular features of individual neurocutaneous syndromes, the relationship between epilepsy and neurodevelopmental outcome across NF1, TSC, and SWS remains incompletely defined. A comparative approach may help clarify which cognitive and behavioural manifestations overlap across syndromes, which features are syndrome-specific, and how epilepsy modifies developmental trajectories in different biological contexts.

**Figure 1:**
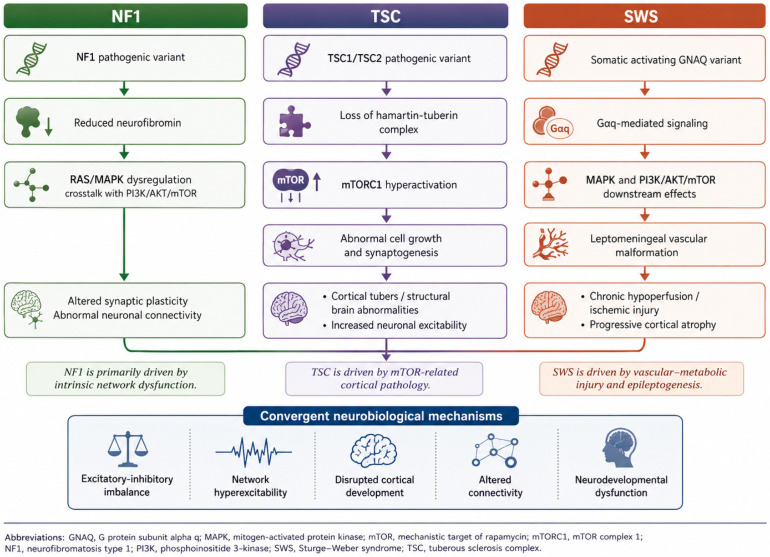
Molecular and mechanistic pathways in NF1, TSC, and Sturge–Weber syndrome. Schematic representation of syndrome-specific molecular alterations and convergent neurobiological mechanisms involved in epileptogenesis and neurodevelopmental dysfunction. NF1, TSC, and Sturge–Weber syndrome originate from distinct upstream defects but converge on altered synaptic plasticity, abnormal cortical or vascular development, excitatory–inhibitory imbalance, network hyperexcitability, and impaired neurodevelopmental trajectories. Arrows indicate the sequential pathogenic cascade within each disorder. Colors distinguish the three conditions: green, neurofibromatosis type 1; purple, tuberous sclerosis complex; orange, Sturge–Weber syndrome. The blue panel summarizes convergent neurobiological mechanisms shared across the three disorders. The graphical layout was created with the assistance of a generative AI tool (ChatGPT, GPT-5.5 Thinking, OpenAI, San Francisco, CA, USA) and subsequently reviewed and edited by the authors.

The relationship between syndrome-specific molecular alterations and convergent neurobiological mechanisms is illustrated in Figure 1.

The aim of this review is to examine the relationship between epilepsy and cognitive and behavioural outcomes in NF1, TSC, and SWS. By integrating clinical, neurodevelopmental, and mechanistic evidence, we explore how epilepsy interacts with syndrome-specific molecular, structural, vascular, and network-level abnormalities to influence cognitive and behavioural trajectories. Emphasis is placed on shared and divergent neurodevelopmental profiles, epilepsy-related prognostic modifiers, clinical monitoring priorities, and future directions for research and individualized care.

## 2. Materials and Methods

**Figure 2:**
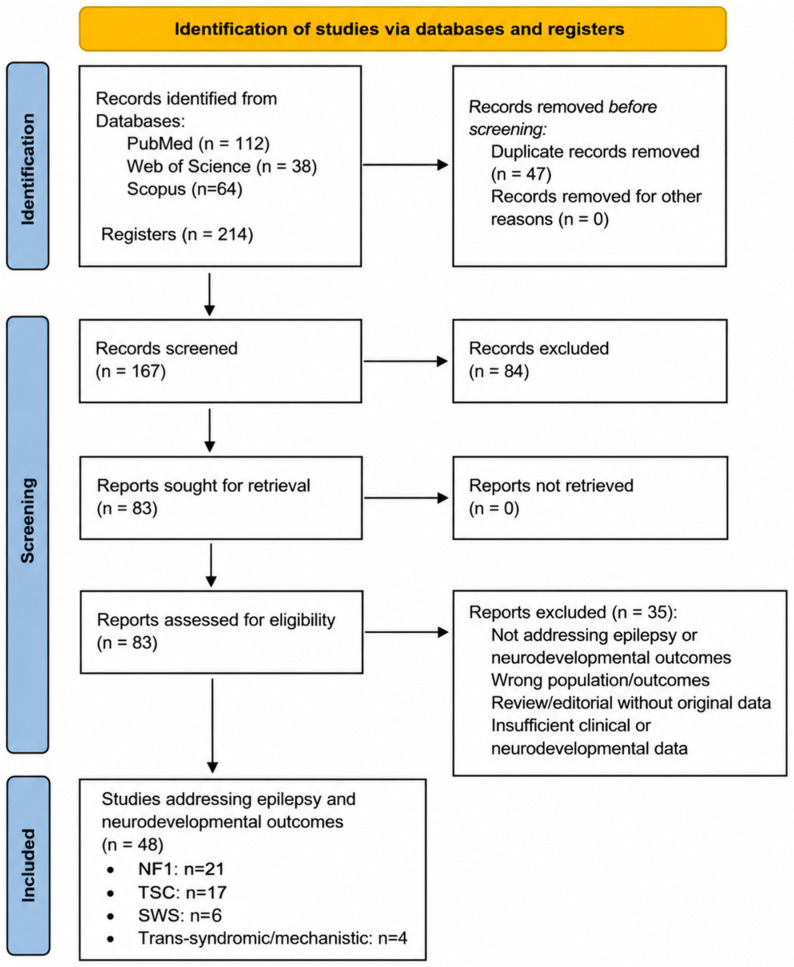
PRISMA 2020 Flow Diagram for the present review adapted from Page et al. [20].

The item selection process is illustrated in the PRISMA 2020 flow diagram (Figure 2). A literature search was performed to identify publications addressing epilepsy, cognitive and behavioural outcomes, and neurodevelopmental dysfunction in the major neurocutaneous syndromes. The search was conducted in PubMed/MEDLINE, Web of Science, and Scopus and covered publications from 2004 to 2025. The search was restricted to human studies, peer-reviewed articles, and publications written in English.

The search strategy combined free-text keywords with controlled vocabulary terms, including Medical Subject Headings (MeSH) where applicable. Boolean operators were used to combine disease-specific, epilepsy-related, and outcome-related terms. The operator OR was used to combine synonyms within the same concept, whereas AND was used to combine different concepts. Search terms included disease-specific terms such as “neurocutaneous syndromes,” “phakomatoses,” “neurofibromatosis type 1,” “tuberous sclerosis complex,” and “Sturge–Weber syndrome”; epilepsy-related terms such as “epilepsy,” “seizures,” “infantile spasms,” and “drug-resistant epilepsy”; and outcome-related terms such as “cognitive impairment,” “intellectual disability,” “neurodevelopmental outcome,” “behavioural disorders,” “executive function,” “attention-deficit/hyperactivity disorder,” and “autism spectrum disorder.” In PubMed/MEDLINE, the search was supplemented with MeSH terms where available, including “Neurofibromatosis 1,” “Tuberous Sclerosis,” “Sturge-Weber Syndrome,” “Epilepsy,” “Seizures,” “Cognition Disorders,” “Intellectual Disability,” “Neurodevelopmental Disorders,” “Behavioural Symptoms,” and “Autism Spectrum Disorder.”

Titles and abstracts were screened for relevance and alignment with the objectives of the review. Full-text articles were subsequently evaluated to extract pertinent clinical, neuropsychological, and mechanistic information. The search yielded a total of 214 articles: 112 from PubMed/MEDLINE, 38 from Web of Science, and 64 from Scopus. After removing 47 duplicates, 167 records were screened, of which 83 were selected for full-text assessment. Ultimately, 48 studies met the inclusion criteria and were incorporated into the qualitative synthesis. Among these, 21 studies focused on NF1, 17 on TSC, 6 on SWS, and 4 provided trans-syndromic or mechanistic perspectives relevant to the review.

The inclusion criteria encompassed human, peer-reviewed studies published in English that investigated epilepsy, cognitive impairment, behavioural dysfunction, neurodevelopmental outcomes, or their underlying molecular mechanisms in NF1, TSC, or SWS, and that reported neuropsychological, clinical, or genetic data regardless of study design. Exclusion criteria included unpublished sources, case reports lacking relevant cognitive or neuropsychological data, animal studies, duplicate records, and articles that did not address the targeted disorders or outcomes. Reference lists of included studies were also screened to identify additional relevant publications.

Data extracted from the selected studies were synthesized qualitatively, with emphasis on the relationship between epilepsy, neurodevelopmental outcomes, and shared molecular mechanisms across NF1, TSC, and SWS. Attention was given to convergent pathways involved in epileptogenesis, neuronal network dysfunction, and cognitive or behavioural impairment.

Although this article was designed as a structured narrative review rather than a systematic review or meta-analysis, the PRISMA 2020 framework was used to document the literature identification, screening, eligibility assessment, and inclusion process. No formal protocol was registered, and no formal methodological quality assessment or formal risk-of-bias evaluation of the included studies was performed.

## 3. Results

### 3.1. Neurodevelopmental Dysfunction in NF1: The Limited Role of Epilepsy

Neurofibromatosis type 1 (NF1) is the most common neurocutaneous syndrome and is frequently associated with cognitive, neuropsychiatric, and neurodevelopmental difficulties that may substantially affect academic achievement, adaptive functioning, and quality of life. Unlike tuberous sclerosis complex (TSC) and Sturge–Weber syndrome (SWS), in which epilepsy is more frequent and more strongly associated with neurodevelopmental outcome, cognitive dysfunction in NF1 commonly occurs independently of seizures and appears to reflect intrinsic abnormalities of neuronal development and network organization [28,29,30,31,32,33,34,35,36,37,38,39,40,41].

Learning difficulties affect approximately 60–80% of individuals with NF1 and represent one of the most common manifestations of the disorder. Cognitive impairment is typically characterized by deficits in attention, working memory, executive functioning, visuospatial processing, and language skills rather than by global intellectual impairment. Although mean intelligence quotient values are generally slightly below population averages, intellectual disability is reported in only a minority of patients, highlighting the heterogeneous nature of the NF1 cognitive phenotype [28,29,30,31].

Executive dysfunction is considered a core feature of NF1-associated neurodevelopmental impairment. Difficulties involving inhibitory control, cognitive flexibility, planning, organization, and working memory have been consistently documented across age groups and are often associated with poorer educational performance and adaptive functioning. Neuroimaging studies have further demonstrated abnormalities in white matter organization and altered fronto-striatal and fronto-parietal connectivity, supporting the concept that cognitive deficits in NF1 arise from disrupted neuronal network development rather than from major structural brain abnormalities [32,33,34,35,36,37,38].

Neuropsychiatric manifestations are also highly prevalent. Attention-deficit/hyperactivity disorder affects more than 40% of individuals with NF1 and represents one of the most frequently reported comorbidities. Autism spectrum traits and social communication difficulties occur at higher rates than in the general population and may further compromise academic performance, social integration, and adaptive behaviour. Emotional and behavioural difficulties often coexist with cognitive deficits, contributing substantially to long-term psychosocial burden and reduced quality of life [35,36,37,38,39,40,41,42].

Overall, studies in NF1 consistently indicate that cognitive and behavioural difficulties may occur independently of epilepsy and are more closely related to intrinsic abnormalities of neuronal signalling, synaptic function, and network connectivity. Epilepsy, when present, may aggravate pre-existing vulnerabilities in attention, learning, executive functioning, and adaptive behaviour, but it should not be interpreted as the principal determinant of the NF1 neurodevelopmental phenotype. This distinction is important when comparing NF1 with TSC and SWS, in which epilepsy is generally more frequent, earlier in onset, and more strongly associated with adverse neurodevelopmental trajectories [29,30,31,37,41].

### 3.2. Epilepsy and Neurodevelopmental Outcomes in Tuberous Sclerosis Complex

Tuberous sclerosis complex (TSC) is a multisystem genetic disorder caused by pathogenic variants in the *TSC1* or *TSC2* genes, leading to dysregulation of the mTOR signalling pathway. Among the major neurocutaneous syndromes, TSC provides one of the clearest examples of the close relationship between epilepsy and neurodevelopmental outcome. Epilepsy affects up to 80–90% of individuals with TSC, often beginning during infancy, and is widely recognized as an important modifier of cognitive, neuropsychiatric, and psychosocial functioning, particularly in interaction with mTOR-related cortical maldevelopment and other disease-related factors [43,44,45,46,47,48].

Neurodevelopmental outcome in TSC is influenced by multiple interacting factors, including genetic background, cortical tuber burden, lesion location, subependymal abnormalities, altered mTOR signalling, epilepsy severity, and treatment response. Accumulating evidence indicates that epilepsy is an important modifier of neurodevelopmental outcome rather than an isolated determinant. Early seizure onset, particularly during infancy, infantile spasms, high seizure burden, prolonged epilepsy duration, and drug-resistant epilepsy have been consistently associated with poorer cognitive performance and a higher risk of intellectual disability [43,44,45,46]. Intellectual disability has been reported in approximately 40–60% of individuals with TSC, ranging from mild learning difficulties to severe cognitive impairment [43,44,45,46]. However, these associations should be interpreted in the broader context of mTOR-related cortical maldevelopment and intrinsic neurodevelopmental vulnerability.

The neuropsychiatric manifestations of TSC are collectively described under the framework of TSC-associated neuropsychiatric disorders (TAND), which encompass cognitive, behavioural, psychiatric, academic, and psychosocial difficulties [45,47]. Even in individuals with preserved intellectual functioning, impairments in attention, executive functioning, language, memory, and academic achievement are frequently observed [43,48]. These findings highlight the complex interaction between underlying structural brain abnormalities, dysregulated mTOR signalling, and epilepsy-related network dysfunction.

Behavioural and psychiatric manifestations are highly prevalent in TSC. Autism spectrum disorder and attention-deficit/hyperactivity disorder occur at substantially higher rates than in the general population, while anxiety, mood disturbances, and social difficulties further contribute to disease burden [45,46,49]. Although these manifestations are multifactorial in origin, several studies suggest that persistent epileptic activity may exacerbate neurodevelopmental abnormalities and negatively influence behavioural outcomes [43,45,50].

The recognition of epilepsy as a major modifier of neurodevelopmental outcome has important clinical implications. Early seizure detection, prompt treatment, and systematic neuropsychological assessment are increasingly regarded as essential components of TSC management. In addition, advances in targeted therapies directed at the mTOR pathway offer new opportunities to address not only seizure control but also broader neurodevelopmental outcomes [44,51,52,53].

Compared with NF1, where cognitive dysfunction often occurs independently of seizures, epilepsy in TSC represents an important contributor to long-term cognitive and behavioural outcome. Its impact is most clinically relevant when seizures begin early, are frequent or prolonged, remain drug-resistant, or coexist with extensive cortical pathology and persistent epileptiform activity.

### 3.3. Epilepsy and Neurodevelopmental Outcomes in Sturge–Weber Syndrome

Sturge–Weber syndrome (SWS) is a rare sporadic neurocutaneous disorder caused by somatic activating mutations in the GNAQ gene, resulting in abnormal vascular development, leptomeningeal angiomatosis, and progressive cerebral involvement [54,55]. Among the major neurocutaneous syndromes, SWS provides another important example of the interaction between epilepsy, structural brain involvement, and neurodevelopmental outcome. Seizures frequently begin during infancy and are often focal, recurrent, and associated with variable neurological and cognitive trajectories [15,54,55].

Early identification of neurological involvement remains a major clinical priority in SWS. Electroencephalography and clinical monitoring may help identify infants at increased risk of epilepsy, while early neurological assessment can support timely intervention and individualized management strategies [56,57,58]. Cognitive impairment in SWS ranges from specific learning difficulties to severe intellectual disability and may evolve over time because of progressive cortical atrophy, chronic hypoperfusion, and recurrent ischemic injury [12,15,59]. Advances in multidisciplinary care, including optimized antiseizure treatment, rehabilitation programs, and, in selected cases, epilepsy surgery, have contributed to improved functional outcomes in affected children [60,61]. Neuroimaging studies have further demonstrated that the extent of leptomeningeal involvement and cerebral perfusion abnormalities correlate with epilepsy severity and cognitive outcome, emphasizing the close interaction between structural brain abnormalities, vascular–metabolic injury, and epileptogenic processes [16,62,63].

Neurodevelopmental outcome in SWS is highly variable and depends largely on the extent of cerebral involvement and epilepsy severity. Studies have shown that seizure onset during the first year of life, frequent seizures, prolonged seizures, status epilepticus, and poor seizure control are associated with lower intellectual functioning and less favourable developmental trajectories [64,65,66]. Conversely, individuals with limited unilateral involvement and well-controlled epilepsy may achieve near-normal cognitive development, suggesting that epilepsy contributes substantially to outcome variability but does not act independently from lesion extent, perfusion impairment, and neurological comorbidity [15,65,66].

Beyond cognitive functioning, behavioural and psychosocial difficulties are increasingly recognized as important components of the SWS phenotype. Children with SWS may experience deficits in attention, executive functioning, emotional regulation, social adaptation, and adaptive functioning, which can significantly affect academic performance and quality of life [12,67,68]. These difficulties often coexist with epilepsy, motor deficits, visual impairment, and neurological comorbidities, creating a cumulative burden that extends beyond seizure control alone.

Autism spectrum features and social communication difficulties may also occur in a substantial subset of patients with SWS and may be more frequent than previously recognized. Studies focusing on behavioural and social communication profiles have reported autism spectrum disorder or clinically significant social communication difficulties in a proportion of affected individuals, indicating that behavioural outcomes in SWS should not be interpreted solely in relation to seizure control or intellectual level [69,70].

Compared with NF1, where epilepsy plays a relatively limited role in shaping cognitive outcome, and similarly to TSC, seizure burden and age at seizure onset represent important modifiers of cognitive and behavioural prognosis in SWS. However, these effects should be interpreted within the broader context of vascular–metabolic brain injury, extent of cerebral involvement, neurological deficits, visual impairment, and access to rehabilitation and educational support.

### 3.4. Shared Mechanisms Linking Epilepsy and Neurodevelopment

Although neurofibromatosis type 1 (NF1), tuberous sclerosis complex (TSC), and Sturge–Weber syndrome (SWS) arise from distinct genetic alterations, increasing evidence suggests that they converge on common neurobiological pathways that contribute to both epileptogenesis and neurodevelopmental dysfunction. Across these disorders, abnormalities in cellular signalling, neuronal connectivity, and network organization may impair cognitive and behavioural development while simultaneously increasing susceptibility to seizures. This mechanistic convergence helps explain the substantial overlap in neurodevelopmental manifestations observed across neurocutaneous syndromes despite their diverse molecular origins [71,72,73].

In NF1, pathogenic variants affecting neurofibromin disrupt RAS/MAPK signalling, leading to alterations in synaptic plasticity, inhibitory neurotransmission, and neuronal network function [22,23,74,75,76,77]. Experimental studies have demonstrated abnormal regulation of GABAergic signalling and impaired cortical inhibitory circuits, mechanisms that have been linked to deficits in learning, attention, and executive functioning [74,77]. These findings support the concept that cognitive dysfunction in NF1 primarily reflects altered neuronal connectivity and network development, with epilepsy playing a comparatively limited role in determining overall neurodevelopmental outcome.

In contrast, dysregulation of the mTOR pathway represents the central pathogenic mechanism in TSC [78]. Hyperactivation of mTOR signalling promotes abnormal neuronal growth, altered cortical architecture, and increased neuronal excitability, creating a biological substrate for both epilepsy and neurodevelopmental impairment [5,44,79,80]. Recent reviews of mTORopathies further support the concept that dysregulated mTOR signalling represents a convergent mechanism linking epilepsy, neurodevelopmental disorders, and emerging targeted therapeutic strategies [81]. In addition, single-cell-based analyses of TSC cortical tubers and focal cortical dysplasia suggest that epileptogenic lesions involve altered cellular interactions and dysregulated cell–cell communication, supporting a network-based view of mTOR-related epileptogenesis [82]. The close association between early-onset epilepsy, drug-resistant seizures, and adverse cognitive outcomes in TSC suggests that epileptic activity may further amplify the effects of underlying structural and molecular abnormalities on the developing brain [43,44,45].

In SWS, somatic activating mutations in *GNAQ* result in abnormal vascular development and leptomeningeal angiomatosis, leading to chronic disturbances in cerebral perfusion and progressive cortical injury [25,54,55,83,84,85]. These abnormalities contribute to the development of epilepsy and may adversely affect cognitive and behavioural functioning through recurrent seizures, impaired network maturation, and ongoing disruption of cortical integrity. The association between seizure burden, age at seizure onset, and neurodevelopmental outcome supports an important contribution of epileptogenesis to disease severity in SWS [65,66].

Despite their distinct molecular origins, these syndromes ultimately converge on a common pathogenic framework characterized by neuronal network dysfunction, increased seizure susceptibility, and neurodevelopmental impairment. The interaction between genetic abnormalities, altered signalling pathways, and epilepsy contributes to a spectrum of cognitive and behavioural outcomes that varies across disorders but shares important mechanistic features. The convergence of these pathways is summarized in Figure 1 and provides a rationale for developing therapeutic strategies that target both seizure control and broader neurodevelopmental outcomes [71,72,73].

### 3.5. Epilepsy-Related Modifiers of Neurodevelopmental Prognosis

Beyond syndrome-specific molecular and structural mechanisms, epilepsy-related variables may further modify cognitive and behavioural prognosis in children with neurocutaneous syndromes. These include age at seizure onset, seizure type, seizure frequency and burden, epilepsy duration, drug resistance, EEG abnormalities, subclinical epileptiform activity, and treatment response. Their relative contribution differs substantially across NF1, TSC, and Sturge–Weber syndrome and should therefore be interpreted in a syndrome-specific context [15,16,43,44,45,55,65,66].

In NF1, epilepsy is usually not the principal determinant of neurodevelopmental outcome. Cognitive and behavioural manifestations are more often related to intrinsic network dysfunction, altered synaptic plasticity, abnormal neuronal connectivity, and RAS/MAPK dysregulation rather than to seizure activity itself [29,30,31,37,41]. However, when epilepsy is present, recurrent seizures, poor seizure control, structural brain lesions, and antiseizure medication burden may aggravate pre-existing vulnerabilities in attention, learning, executive function, and adaptive functioning [29,31,37,41].

In TSC, epilepsy-related factors appear to have a stronger influence on neurodevelopmental prognosis than in NF1. Early seizure onset, particularly during infancy, infantile spasms, high seizure burden, prolonged epilepsy duration, drug-resistant epilepsy, and persistent epileptiform abnormalities have been associated with a higher risk of intellectual disability, autism spectrum disorder, executive dysfunction, impaired adaptive functioning, and TSC-associated neuropsychiatric disorders [43,44,45,49,50]. These effects are amplified by mTOR-related cortical pathology, including cortical tubers, abnormal synaptogenesis, altered neuronal excitability, and disrupted connectivity [44,51,52,53]. Consequently, seizure control in TSC should be considered not only a neurological objective but also a neurodevelopmental priority.

In Sturge–Weber syndrome, the prognostic role of epilepsy is closely linked to the extent of cerebral involvement and vascular–metabolic injury. Early-onset focal seizures, frequent or prolonged seizures, status epilepticus, drug resistance, and persistent EEG abnormalities may contribute to cognitive impairment, developmental slowing, executive dysfunction, and reduced adaptive functioning [15,16,55,65,66]. In this disorder, epilepsy should be interpreted as part of a dynamic interaction between leptomeningeal angiomatosis, impaired perfusion, progressive cortical injury, and network hyperexcitability [15,16,54,55,65,66].

**Figure 3:**
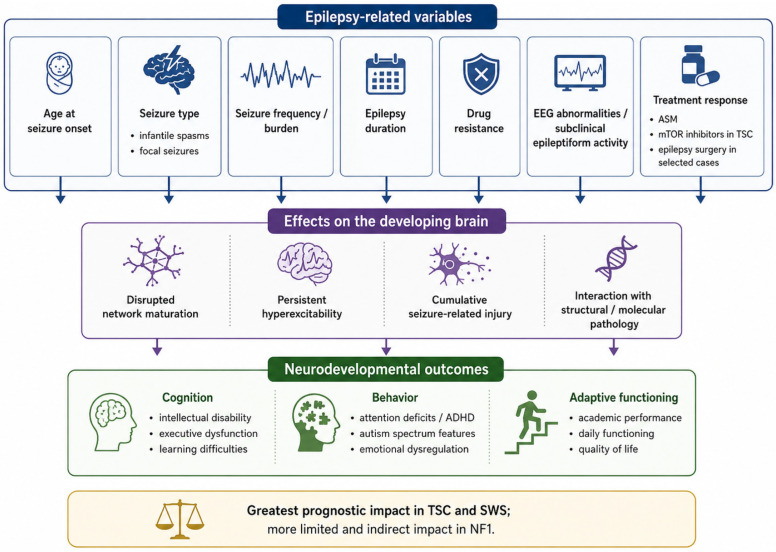
Epilepsy-related modifiers of neurodevelopmental prognosis. The figure summarizes epilepsy-related variables, their effects on the developing brain, and their impact on neurodevelopmental outcomes. Arrows indicate the directional relationship from epilepsy-related clinical variables to intermediate effects on brain development and subsequent neurodevelopmental outcomes. Colors distinguish the main levels of the framework: blue, epilepsy-related variables; purple, effects on the developing brain; green, neurodevelopmental outcomes; yellow, overall prognostic interpretation. The graphical layout was created with the assistance of ChatGPT (GPT-5.5 Thinking, OpenAI, San Francisco, CA, USA) and subsequently reviewed and edited by the authors.

Treatment-related factors also require careful interpretation. Persistent seizures and drug resistance are associated with poorer neurodevelopmental outcomes, whereas early seizure recognition, effective antiseizure therapy, targeted treatment in selected patients, and epilepsy surgery in carefully selected cases may modify developmental trajectories [44,51,52,53,56,60,63]. At the same time, antiseizure medication burden, sedative effects, polytherapy, and timing of treatment may influence attention, learning, behaviour, and adaptive functioning. The main epilepsy-related modifiers of neurodevelopmental prognosis across NF1, TSC, and Sturge–Weber syndrome are summarized in Figure 3.

### 3.6. Comparative Overview of Cognitive and Behavioural Outcomes Across Neurocutaneous Syndromes

Although NF1, TSC, and SWS are all associated with neurodevelopmental impairment, the clinical pattern and main determinants of cognitive, emotional, and adaptive difficulties differ across disorders. Shared features include attention difficulties, executive dysfunction, learning problems, dysregulation of behavior, social communication difficulties, impaired adaptive functioning, and psychosocial burden. However, the dominant profiles diverge. NF1 is characterized mainly by attention, executive, visuospatial, and learning difficulties, often in the absence of epilepsy. TSC is more frequently associated with intellectual disability, autism spectrum disorder, TSC-associated neuropsychiatric disorders, early-onset epilepsy, infantile spasms, and drug-resistant epilepsy. In SWS, cognitive and functional outcomes reflect the combined influence of vascular brain involvement, focal epilepsy, motor and visual impairment, cognitive variability, and social communication or adaptive difficulties [29,31,43,45,55,65,66,67,68,69,70].

The cognitive profiles associated with these syndromes also differ considerably. Executive dysfunction and attention deficits are particularly prominent in NF1, whereas TSC is more frequently associated with intellectual disability, autism spectrum disorder, and broader TSC-associated neuropsychiatric disorders (TAND). In SWS, neurodevelopmental outcomes are highly variable and largely influenced by the extent of cerebral involvement and epilepsy severity. Despite these differences, all three conditions may significantly affect educational attainment, adaptive functioning, psychosocial well-being, and quality of life [37,41,45,48,67,68].

Recent evidence suggests that distinct genetic and molecular abnormalities ultimately converge on common mechanisms involving neuronal network dysfunction, altered connectivity, and disrupted neurodevelopment. These shared pathways may explain the overlapping cognitive and behavioural manifestations observed across neurocutaneous syndromes while also highlighting syndrome-specific factors that influence prognosis and therapeutic response [71,72,73].

**Figure 4:**
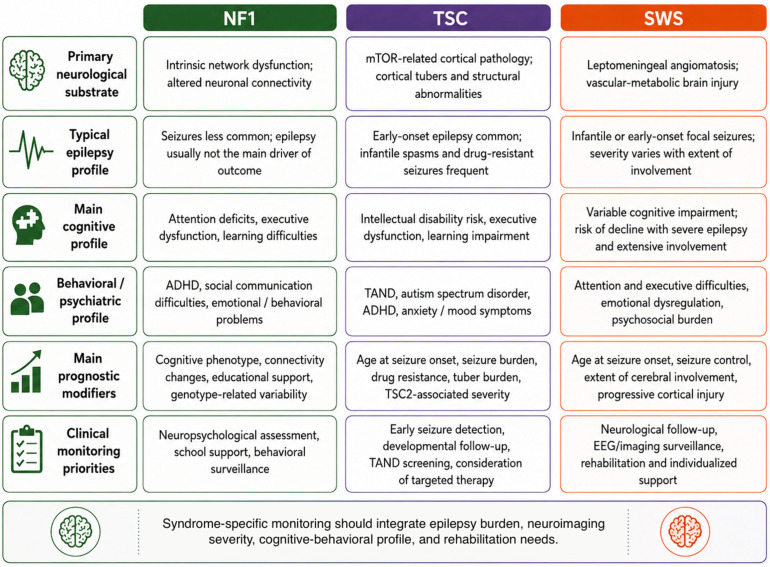
Comparative clinical monitoring and prognostic framework across NF1, TSC, and SWS. Schematic comparison of neurological substrates, epilepsy profiles, cognitive and behavioural manifestations, prognostic modifiers, and monitoring priorities across the three disorders. The figure emphasizes the need for syndrome-specific follow-up integrating epilepsy burden, neuroimaging severity, cognitive-behavioural phenotype, and individualized rehabilitation needs. The graphical layout was created with the assistance of a generative AI tool and subsequently reviewed and edited by the authors.

The main clinical and prognostic differences between NF1, TSC, and Sturge–Weber syndrome are summarized in Figure 4. This comparative framework highlights the distinct neurological substrates, epilepsy profiles, cognitive and behavioural manifestations, prognostic modifiers, and monitoring priorities across the three disorders.

To further support the cross-syndrome comparison and reduce repetition, the key clinical, epilepsy-related, cognitive, behavioural, and prognostic features are detailed in Table 1 and Table 2.

Taken together, these findings support a spectrum-based view of neurocutaneous syndromes in which disorder-specific genetic alterations converge on shared neurodevelopmental pathways but differ in the relative contribution of epilepsy to long-term functional outcomes. While epilepsy contributes substantially to prognosis in TSC and SWS, cognitive difficulties and adaptive dysfunction in NF1 appear to be more directly related to intrinsic abnormalities of neuronal development and network organisation. Recognition of these similarities and differences may facilitate earlier risk stratification, individualized neuropsychological monitoring, and the development of targeted therapeutic strategies aimed at improving long-term outcomes in affected children [71,72,73].

## 4. Discussion

This review examined the relationship between epilepsy and cognitive and behavioural outcomes across three major neurocutaneous syndromes: neurofibromatosis type 1 (NF1), tuberous sclerosis complex (TSC), and Sturge–Weber syndrome (SWS). Although these disorders arise from distinct genetic mechanisms, they share important neurodevelopmental consequences and show substantial overlap in cognitive, neuropsychiatric, and adaptive manifestations. Our findings indicate that epilepsy contributes substantially to neurodevelopmental outcome in TSC and SWS, whereas its contribution appears more limited in NF1, where cognitive dysfunction is primarily related to intrinsic abnormalities of neuronal development and network organization [71,72,73].

A central observation emerging from the reviewed literature is the heterogeneous role of epilepsy across neurocutaneous syndromes. In TSC and SWS, early seizure onset, high seizure burden, and drug-resistant epilepsy are consistently associated with poorer cognitive performance, intellectual disability, and impaired adaptive functioning. These findings support the concept that epileptic activity occurring during critical periods of brain maturation may interfere with neurodevelopmental processes and exacerbate the effects of underlying structural and molecular abnormalities [43,44,45,55,65,66]. In contrast, individuals with NF1 frequently exhibit attention deficits, executive dysfunction, learning difficulties, and social communication problems even in the absence of epilepsy, suggesting that neurodevelopmental impairment is largely driven by syndrome-specific alterations in neuronal signalling and connectivity rather than by seizure-related mechanisms [29,30,31,37,41].

Across the three disorders, several neurodevelopmental characteristics overlap, including attention difficulties, executive dysfunction, learning problems, behavioural dysregulation, social communication difficulties, adaptive functioning impairment, and psychosocial burden. However, the dominant profiles diverge. NF1 is more commonly characterized by attention, executive, visuospatial, and learning difficulties, often in the absence of epilepsy. TSC is more strongly associated with intellectual disability, autism spectrum disorder, TSC-associated neuropsychiatric disorders, early-onset epilepsy, infantile spasms, and drug-resistant epilepsy. In SWS, neurodevelopmental outcome reflects a distinctive interaction between vascular brain involvement, focal epilepsy, motor and visual impairment, cognitive variability, and social communication or behavioural difficulties. Taken together, these findings suggest that epilepsy should be interpreted as part of a broader syndrome-specific neurodevelopmental context rather than as an isolated explanation for cognitive and behavioural impairment.

Despite these differences, increasing evidence supports the existence of shared neurobiological pathways linking epilepsy and neurodevelopmental dysfunction. Dysregulation of RAS/MAPK signalling in NF1, mTOR hyperactivation in TSC, and GNAQ-mediated vascular abnormalities in SWS ultimately converge on disrupted neuronal network function, altered synaptic plasticity, and impaired brain connectivity [71,72,73]. These convergent mechanisms may explain why cognitive and behavioural difficulties occur across all three disorders despite their distinct molecular origins. The concept of network dysfunction provides a useful framework for understanding the interaction between genetic abnormalities, epileptogenesis, and neurodevelopmental outcomes in neurocutaneous syndromes.

The findings of this review have important clinical implications. First, they emphasize the need for routine neurodevelopmental surveillance in all affected children, regardless of seizure status. While seizure control remains a major therapeutic priority in TSC and SWS, neuropsychological assessment should not be limited to patients with epilepsy. Early identification of cognitive, behavioural, and adaptive difficulties may facilitate timely intervention and improve long-term educational and psychosocial outcomes. Second, the marked influence of epilepsy on neurodevelopmental trajectories in TSC and SWS highlights the importance of early diagnosis and aggressive seizure management, particularly during infancy and early childhood, when the developing brain may be most vulnerable to epileptic activity [44,63].

Recent advances in targeted therapies further support a precision medicine approach to neurocutaneous syndromes. In TSC, mTOR inhibitors have expanded therapeutic options beyond conventional antiseizure medications and may offer broader benefits through modification of disease-related pathways [44,51,86]. Similarly, ongoing research into signalling pathways involved in NF1 and SWS may facilitate the development of novel interventions aimed not only at seizure control but also at improving cognitive and behavioural outcomes. Future studies should explore whether early pathway-specific interventions can alter neurodevelopmental trajectories and reduce long-term disability [71,72,73].

### 4.1. Clinical Implications and Individualized Monitoring

The findings of this review support a syndrome-specific approach to clinical monitoring and intervention. Although NF1, TSC, and Sturge–Weber syndrome all require multidisciplinary follow-up, the priorities differ according to the dominant mechanisms, epilepsy profile, neuroimaging findings, and cognitive-behavioural phenotype.

In NF1, clinical surveillance should focus primarily on early recognition of cognitive, behavioural, and educational difficulties, even in the absence of epilepsy. Periodic neuropsychological assessment should evaluate attention, executive function, visuospatial abilities, language, learning profile, adaptive functioning, and social communication. Screening for ADHD, autism spectrum traits, anxiety, emotional dysregulation, and school-related difficulties should be integrated into follow-up [8,9,10]. Educational support, behavioural interventions, and family counselling are central components of care. EEG assessment and epilepsy-directed investigations should be guided by clinical suspicion rather than performed solely because of the NF1 diagnosis.

In TSC, monitoring should begin early in infancy and should integrate seizure surveillance with developmental follow-up. Because early-onset seizures, infantile spasms, high seizure burden, drug-resistant epilepsy, and persistent epileptiform activity are major predictors of adverse outcome, early EEG monitoring, prompt recognition and treatment of epileptic spasms, and rapid optimization of seizure control are clinically important [43,44,45]. Neurodevelopmental surveillance should include systematic screening for TSC-associated neuropsychiatric disorders, intellectual disability, autism spectrum disorder, ADHD, sleep problems, anxiety, behavioural dysregulation, academic difficulties, and adaptive functioning impairment [49,50]. Targeted therapies may be considered in selected patients according to current clinical recommendations and individualized risk–benefit assessment [44,51,52,53]. Epilepsy surgery may also be considered in carefully selected patients with drug-resistant epilepsy after multidisciplinary evaluation [87].

In Sturge–Weber syndrome, clinical monitoring should combine seizure management with neuroimaging-based assessment of cerebral involvement and rehabilitation planning. Children with early-onset seizures, extensive leptomeningeal involvement, recurrent stroke-like episodes, hemiparesis, visual impairment, or refractory epilepsy require close neurological and developmental follow-up. EEG and neuroimaging surveillance may help identify patients at higher risk of neurological deterioration. Rehabilitation, physiotherapy, occupational therapy, visual assessment, school support, and psychological care should be individualized according to motor deficits, visual involvement, cognitive profile, seizure control, and adaptive functioning [60]. In selected patients with refractory epilepsy, epilepsy surgery may be considered after multidisciplinary evaluation, with attention to seizure control, motor outcome, cognitive trajectory, and rehabilitation needs [88].

Across all three syndromes, intervention should not be delayed until global developmental impairment becomes evident. Early cognitive and behavioural screening, individualized educational support, seizure optimization, family-centred counselling, and coordinated care involving paediatric neurology, genetics, neuropsychology, psychiatry, rehabilitation, ophthalmology, dermatology, and educational specialists may improve long-term functioning. Future care models should move from general surveillance toward risk-stratified monitoring based on syndrome, molecular mechanism, epilepsy profile, neuroimaging severity, and developmental trajectory.

### 4.2. Limitations

Several limitations should be acknowledged.

First, although the literature selection process was documented using the PRISMA 2020 framework, this article was designed as a structured narrative review rather than a systematic review or meta-analysis. No protocol was prospectively registered, and no formal risk-of-bias or methodological quality assessment was performed. Therefore, the strength of evidence could not be graded, and the conclusions should be interpreted as a qualitative synthesis rather than as evidence derived from a systematic quantitative analysis.

Second, the search strategy combined free-text terms with controlled vocabulary terms, including MeSH terms where applicable. However, differences in indexing across databases and the use of database-specific terminology may still have led to missed studies. This is particularly relevant for papers using alternative terminology for neurocutaneous syndromes, epilepsy-related outcomes, neurodevelopmental impairment, or behavioural manifestations.

Third, the review was restricted to English-language publications. This restriction may have introduced language bias by excluding relevant studies published in other languages. As a result, the synthesis may overrepresent data from English-language literature and from healthcare systems or research groups with higher international publication visibility.

Fourth, the selected time frame, from 2004 to 2025, may have excluded older studies that contributed to the historical understanding of epilepsy and neurodevelopmental outcomes in NF1, TSC, or Sturge–Weber syndrome. Conversely, because the field is rapidly evolving, especially regarding targeted therapies, advanced neuroimaging, molecular profiling, and early intervention strategies, some recent findings may not yet have been fully replicated or incorporated into consensus recommendations.

Fifth, case reports and small reports without relevant neuropsychological, developmental, or mechanistic data were excluded. This decision was intended to reduce anecdotal interpretation and to focus on broader clinical and mechanistic patterns. However, it may have led to underrepresentation of rare phenotypes, atypical epilepsy trajectories, severe presentations, or early observations regarding emerging targeted treatments.

Sixth, the included studies were heterogeneous in terms of study design, patient age, disease severity, epilepsy definitions, seizure classification, neuropsychological instruments, imaging protocols, treatment exposure, and follow-up duration. This heterogeneity limited direct comparison across NF1, TSC, and Sturge–Weber syndrome and precluded meta-analysis or pooled estimates. Stratified data by age group, seizure type, epilepsy duration, genotype, lesion burden, neuroimaging severity, and treatment response were not consistently available across studies.

Seventh, cognitive and behavioural outcomes in neurocutaneous syndromes are multifactorial. They may be influenced by genetic background, structural brain abnormalities, epilepsy severity, antiseizure medication exposure, targeted therapies, educational support, socioeconomic factors, access to early intervention, and availability of multidisciplinary care. Most available studies do not fully adjust for all these variables, making it difficult to separate the independent contribution of epilepsy from the underlying molecular, structural, vascular, and environmental determinants of neurodevelopmental outcome.

Finally, direct comparative studies examining NF1, TSC, and Sturge–Weber syndrome within the same methodological framework remain scarce. Most evidence comes from syndrome-specific cohorts using different outcome measures and follow-up strategies. Therefore, the cross-syndrome comparisons proposed in this review should be interpreted as clinically oriented and hypothesis-generating. Future multicentre longitudinal studies using standardized epilepsy, neuroimaging, neuropsychological, behavioural, and functional outcome measures are needed to clarify syndrome-specific and shared predictors of long-term prognosis.

### 4.3. Future Directions

Future research should focus on longitudinal studies integrating neuroimaging, electrophysiological, genetic, and neuropsychological data to better define predictors of neurodevelopmental outcome. Improved understanding of the mechanisms linking epilepsy, neuronal network dysfunction, and cognitive impairment may facilitate earlier risk stratification and the development of individualized therapeutic strategies. In TSC, this direction is illustrated by the PROTECT trial protocol, which evaluates the long-term neuropsychological outcome of pre-emptive mTOR inhibitor treatment initiated in infants diagnosed with TSC under 4 months of age [89,90]. In parallel, greater attention should be directed toward quality of life, adaptive functioning, educational support, and psychosocial well-being, as these factors ultimately determine long-term outcomes for affected children and their families [91,92].

Overall, the evidence reviewed here supports a model in which epilepsy acts as an important modifier of neurodevelopmental outcome, particularly in TSC and SWS, where seizures are more frequent, earlier in onset, and more often treatment resistant. However, epilepsy should be interpreted in interaction with syndrome-specific molecular, structural, vascular, and network-level abnormalities rather than as an isolated determinant of cognitive and behavioural dysfunction. Recognising both the shared and syndrome-specific mechanisms underlying neurodevelopmental impairment may contribute to more accurate prognostic assessment and more comprehensive, patient-centred care in neurocutaneous syndromes.

## 5. Conclusions

Neurofibromatosis type 1 (NF1), tuberous sclerosis complex (TSC), and Sturge–Weber syndrome (SWS) are distinct neurocutaneous syndromes that share a substantial burden of cognitive, behavioural, and neurodevelopmental impairment. Although their underlying genetic, molecular, structural, and vascular mechanisms differ, all three disorders involve disruptions of neuronal network development and function that may contribute to adverse neurodevelopmental outcomes. The evidence reviewed in this study indicates that the contribution of epilepsy to these outcomes varies considerably across syndromes.

In TSC and SWS, early seizure onset, high seizure burden, drug-resistant epilepsy, and poor seizure control may compound pre-existing neurodevelopmental vulnerability and are associated with less favourable cognitive and behavioural trajectories. In NF1, cognitive and behavioural dysfunction appears to be driven more strongly by intrinsic abnormalities of neuronal signalling, synaptic function, and connectivity, with epilepsy playing a comparatively limited but potentially aggravating role in affected individuals. Understanding these syndrome-specific differences, while recognizing shared mechanisms linking epileptogenesis and neurodevelopmental dysfunction, may improve prognostic assessment, support earlier neuropsychological intervention, and facilitate individualized therapeutic strategies aimed at optimizing long-term outcomes and quality of life in affected children.

## Figures and Tables

**Table 1:** Comparative clinical, epilepsy-related, cognitive, and behavioural profiles in NF1, TSC, and Sturge–Weber syndrome. The table summarizes evidence discussed in the corresponding sections of the manuscript regarding NF1 cognitive and behavioural manifestations, TSC epilepsy and TSC-associated neuropsychiatric disorders, and Sturge–Weber syndrome epilepsy, cognitive, behavioural, social communication, and adaptive functioning outcomes. [8,9,10,11,12,13,14,15,16,43,44,45,46,47,48,49,50,55,64,65,66,67,68,69,70].

Domain	NF1	TSC	Sturge–Weber Syndrome
Primary pathogenic mechanism	Germline or mosaic *NF1* pathogenic variants leading to reduced neurofibromin function and RAS/MAPK dysregulation	*TSC1* or *TSC2* pathogenic variants leading to loss of hamartin–tuberin complex function and mTORC1 hyperactivation	Somatic activating *GNAQ* variants leading to Gαq-mediated signalling abnormalities and leptomeningeal vascular malformations
Main neurological substrate	Intrinsic network dysfunction, altered synaptic plasticity, abnormal neuronal connectivity	mTOR-related cortical maldevelopment, cortical tubers, abnormal synaptogenesis, altered neuronal excitability	Leptomeningeal angiomatosis, impaired perfusion, ischemic injury, calcification, progressive cortical atrophy
Epilepsy frequency and relevance	Seizures are less frequent than in TSC and SWS; epilepsy is usually not the principal driver of cognitive outcome	Epilepsy is very common and often begins in infancy; prognosis is influenced by early onset, seizure burden, drug resistance, and cortical pathology.	Epilepsy is common, often early-onset and focal; prognosis is strongly influenced by seizure burden and extent of cerebral involvement
**Typical seizure profile**	Variable; focal seizures may occur, often requiring evaluation for associated structural lesions or comorbidities	Infantile spasms, focal seizures, multifocal epilepsy, and drug-resistant epilepsy are frequent	Early-onset focal seizures, prolonged seizures, status epilepticus, and recurrent seizures may occur
Epilepsy-related prognostic modifiers	Seizure frequency, structural brain lesions, drug resistance, medication burden, coexisting cognitive vulnerabilities	Age at seizure onset, infantile spasms, seizure burden, epilepsy duration, drug resistance, EEG abnormalities, treatment response	Age at seizure onset, seizure frequency, seizure control, status epilepticus, extent of leptomeningeal involvement, perfusion abnormalities
Cognitive profile	Attention deficits, executive dysfunction, learning difficulties, visuospatial impairment, language-related difficulties; intellectual disability is less common	Intellectual disability risk, global developmental delay, executive dysfunction, language impairment, learning difficulties; wide interindividual variability	Variable cognitive impairment; risk of developmental slowing or decline in patients with early severe epilepsy or extensive cerebral involvement
Behavioral and psychiatric profile	ADHD, social communication difficulties, autism spectrum traits, emotional and behavioural problems	TAND, autism spectrum disorder, ADHD, anxiety, mood symptoms, sleep problems, behavioural dysregulation	Attention difficulties, executive dysfunction, autism spectrum features, social communication difficulties, emotional dysregulation, psychosocial burden, and adaptive difficulties
Main determinant of neurodevelopmental outcome	Intrinsic neurodevelopmental and connectivity abnormalities; epilepsy usually has limited or indirect contribution	Interaction between mTOR-related cortical pathology and early, severe, or drug-resistant epilepsy	Interaction between vascular–metabolic brain injury, extent of cerebral involvement, and epilepsy burden
Clinical monitoring priorities	Neuropsychological assessment, ADHD/autism spectrum screening, school support, behavioural surveillance; EEG when clinically indicated	Early EEG and seizure surveillance, prompt treatment of infantile spasms, TAND screening, developmental follow-up, consideration of targeted therapy	Neurological follow-up, EEG and neuroimaging surveillance, seizure optimization, rehabilitation, visual assessment, individualized educational support

**Table 2:** Stratified predictors of adverse cognitive and behavioural outcomes across NF1, TSC, and Sturge–Weber syndrome. The table summarizes evidence discussed in the corresponding sections of the manuscript regarding NF1 cognitive and behavioural manifestations, TSC epilepsy and TAND, and Sturge–Weber syndrome epilepsy and neurodevelopmental outcomes [8,9,10,15,16,43,44,45,49,50,55,65,66].

Predictor Category	NF1	TSC	Sturge–Weber Syndrome
Age-related factors	Difficulties may become more evident during preschool and school years, especially when executive and academic demands increase	Infancy and early childhood represent high-risk periods because early-onset seizures and infantile spasms may disrupt critical developmental windows	Early infancy is high risk when seizures begin early and cerebral involvement is extensive
Epilepsy onset	Usually not the main determinant, but early-onset or recurrent seizures may worsen pre-existing vulnerabilities	Early seizure onset is strongly associated with poorer cognitive and behavioural outcomes	Seizure onset during infancy is associated with less favourable cognitive and neurological trajectories
Seizure type	Focal seizures may occur; prognostic relevance depends on frequency, control, and associated lesions	Infantile spasms and focal/multifocal seizures are important predictors of developmental risk	Focal seizures, prolonged seizures, and status epilepticus may indicate higher neurological risk
Seizure burden and epilepsy duration	Usually, lower prognostic weight than in TSC and SWS, but persistent seizures may aggravate attention, learning, and adaptive functioning	High seizure burden, prolonged uncontrolled epilepsy, and drug resistance are major predictors of intellectual disability, ASD, and TAND	Frequent seizures, recurrent prolonged seizures, and poor seizure control are associated with cognitive impairment and possible decline
EEG abnormalities	EEG guided by clinical suspicion; persistent epileptiform activity may require individualized interpretation	Epileptiform abnormalities, especially when early or persistent, may support risk stratification and early intervention	EEG may help identify seizure risk and monitor epileptiform activity in patients with cerebral involvement
Neuroimaging severity	White matter abnormalities, altered connectivity, unidentified bright objects, or structural lesions may contribute to cognitive phenotype	Cortical tuber burden, tuber location, subependymal lesions, white matter abnormalities, and epileptogenic networks influence outcome	Extent of leptomeningeal angiomatosis, perfusion abnormalities, cortical atrophy, calcification, and hemispheric involvement influence prognosis
Molecular/genotype-related factors	Genotype-specific effects may influence cognitive phenotype; RAS/MAPK dysregulation affects synaptic and network function	*TSC2* variants and severe mTOR pathway dysregulation are often associated with more severe disease	Somatic mosaicism and distribution of vascular involvement influence phenotype severity
Treatment-related factors	Antiseizure medication burden may affect attention or learning in some patients; educational and behavioural interventions are central	Early seizure control, treatment of infantile spasms, mTOR inhibitors, and epilepsy surgery in selected cases may modify trajectory	Seizure control, prevention of prolonged seizures, rehabilitation, and surgery in selected refractory cases may improve functional outcome
Behavioural modifiers	ADHD, executive dysfunction, social communication difficulties, anxiety, and educational support influence long-term adaptation	TAND manifestations, ASD, ADHD, sleep problems, anxiety, behavioural dysregulation, and family support influence functioning	Emotional dysregulation, motor impairment, visual deficits, school adaptation, and psychosocial support influence quality of life
Evidence gaps	Need for longitudinal studies linking genotype, imaging, cognition, behaviours, and epilepsy status	Need for long-term studies on pre-emptive treatment, targeted therapies, neuropsychological outcomes, and multicentred risk models	Need for longitudinal neuroimaging, seizure trajectory studies, rehabilitation outcomes, and multicentre cohorts

## Data Availability

No new data were created or analysed in this study. Data sharing is not applicable to this article.

## Abbreviations

The following abbreviations are used in this manuscript:

ADHD	attention-deficit/hyperactivity disorder
AKT	protein kinase B
ASM	antiseizure medication
ASD	autism spectrum disorder
EEG	electroencephalography
GABA	gamma-aminobutyric acid
Gαq	G protein alpha q subunit
GNAQ	G protein subunit alpha q
MAPK	mitogen-activated protein kinase
mTOR	mechanistic target of rapamycin
mTORC1	mechanistic target of rapamycin complex 1
NCS	neurocutaneous syndromes
NF1	neurofibromatosis type 1
PI3K	phosphoinositide 3-kinase
PRISMA	Preferred Reporting Items for Systematic Reviews and Meta-Analyses
RAS	rat sarcoma family of small GTPases
SWS	Sturge–Weber syndrome
TAND	TSC-associated neuropsychiatric disorders
TSC	tuberous sclerosis complex
TSC1	TSC complex subunit 1
TSC2	TSC complex subunit 2
